# Galactic cosmic ray effects on iron and nickel isotopes in iron meteorites

**DOI:** 10.1111/maps.13446

**Published:** 2020-02-11

**Authors:** David L. Cook, Ingo Leya, Maria Schönbächler

**Affiliations:** ^1^ Institute for Geochemistry and Petrology ETH Zürich Clausiusstrasse 25 8092 Zürich Switzerland; ^2^ Space Research and Planetology University of Bern Sidlerstrasse 5 3012 Bern Switzerland; ^3^ Present address: Earth Observatory of Singapore 50 Nanyang Avenue Singapore 639798 Singapore

## Abstract

We present model calculations for cosmogenic production rates in order to quantify the potential effects of spallation and neutron capture reactions on Fe and Ni isotopes in iron meteorites. We aim to determine whether the magnitude of any cosmogenic effects on the isotopic ratios of Fe and/or Ni may hinder the search for nucleosynthetic variations in these elements or in the application of the ^60^Fe‐^60^Ni chronometer. The model shows that neutron capture reactions are the dominant source of shifts in Fe and Ni isotopic ratios and that spallation reactions are mostly negligible. The effects on ^60^Ni are sensitive to the Co/Ni ratio in the metal. The total galactic cosmic ray (GCR) effects on ^60^Ni and ^64^Ni can be minimized through the choice of normalizing isotopes (^61^Ni/^58^Ni versus ^62^Ni/^58^Ni). In nearly all cases, the GCR effects (neutron capture and/or spallation) on Fe and Ni isotopic ratios are smaller than the current analytical resolution of the isotopic measurements. The model predictions are compared to the Fe and Ni isotopic compositions measured in a suite of six group IAB irons with a range of cosmic ray exposure histories. The experimental data are in good agreement with the model results. The minimal effects of GCRs on Fe and Ni isotopes should not hamper the search for nucleosynthetic variations in these two elements or the application of the ^60^Fe‐^60^Ni chronometer in iron meteorites or chondrites.

## Introduction

The solar system formed from a cloud of material comprised of gas and dust that originated in multiple stellar sources (e.g., supernovae, red giants). The isotopic record in meteorites can be used to constrain these stellar sources, the physical processes that operated in the protoplanetary disk (e.g., mixing), and possibly the environment from which the solar system initially formed (e.g., Cameron and Truran 1977; Boss 2017; Dwarkadas et al. 2017). In addition, isotopic studies that utilize chronometers based on short‐lived radionuclides (e.g., ^26^Al, ^60^Fe, ^182^Hf) provide a means to elucidate the time line for events in the early solar system (e.g., Davis and McKeegan 2014). However, prior to their arrival on Earth, meteoroids are subjected to bombardment by solar cosmic rays (SCRs) and galactic cosmic rays (GCRs) in outer space. Effects from SCRs are limited to the outermost portions of the meteoroids, whereas the mean penetration depth for GCRs is in the range of 50 cm and affects all parts of the meteoroid. Thus, most cosmogenic nuclide studies focus on the effects induced by GCRs. Interactions with GCRs can lead to nuclear reactions that alter the original isotopic composition of the target material, and these effects are well documented in meteorites (e.g., Masarik and Reedy 1994; Masarik 1997; Leya et al. 2003; Leya and Masarik 2013). Because they are non‐mass‐dependent, GCR effects may masquerade as nucleosynthetic isotopic variations and/or may obscure the true age of samples. In the case of iron meteorites, GCR effects have been reported for various stable isotopes, including Ru **(**Fischer‐Gödde et al. 2015), Os (e.g., Walker 2012; Wittig et al. 2013), Pt (e.g., Kruijer et al. 2013; Wittig et al. 2013; Hunt et al. 2017), Re (Liu et al. 2017), Cr (Liu et al. 2019), and Cu (Chen et al. 2016)**.** Effects from GCRs have also been shown to affect chronometers in iron meteorites, including ^107^Pd‐^107^Ag (Matthes et al. 2015) and ^182^Hf‐^182^W (e.g., Markowski et al. 2006; Kruijer et al. 2013; Hunt et al. 2018).

Small variations in Ni isotopes ascribed to a nucleosynthetic origin have been reported in the metal phase of some iron meteorites (Steele et al. 2011; Nanne et al. 2019). Iron meteorites have also been used to constrain the initial solar system abundance of ^60^Fe (Cook et al. 2006; Dauphas et al. 2008). Recently, small Fe isotopic variations were reported in troilite inclusions from iron meteorites (Cook and Schönbächler 2017), but their origin is unclear. It is possible that the above variations have a component related to GCR effects. Models for GCR‐induced isotopic effects on multiple elements in iron meteorites have been developed (e.g., Leya and Masarik 2013), and previous studies showed that GCR effects can be accounted for and corrected using a combination of several isotopic ratios with GCR models (e.g., Kruijer et al. 2013; Fischer‐Gödde et al. 2015; Matthes et al. 2015; Cook et al. 2018; Hunt et al. 2018). Currently, a systematic treatment of potential GCR effects on Fe and Ni isotopes is lacking. Here, we present a model to quantify the GCR effects on Fe and Ni isotopes that is based on the work of Leya and Masarik (2013). The effects include both spallation and neutron capture reactions for iron meteoroids with various radii, and the magnitude of the effects as a function of depth is explored.

In addition to the modelling, a suite of six group IAB irons were selected to study the possible GCR effects on Fe and Ni isotopic ratios. These six samples span a range of exposure histories with cosmic ray exposure (CRE) ages from 5 to 875 Ma (Hunt et al. [2018] and references therein). Nucleosynthetic variations in isotopic ratios appear to be absent in IAB irons (e.g., Chen et al. 2010; Burkhardt et al. 2011; Fischer‐Gödde et al. 2015) despite being present in various other groups of iron meteorites. The absence of such effects makes the IAB irons ideally suited to study GCR effects. In addition, Pt isotopes were previously measured in the same samples (Hunt et al. 2018), which can be used as an independent monitor of GCR effects and can be used to validate the GCR model predictions for effects on Fe and Ni isotopes.

## Methods

### Samples and Preparation

Six IAB irons were chosen to sample a range of cosmic ray exposure histories. The sample preparation and digestion are described by Hunt et al. (2018) for a parallel project on Pt‐W isotopes. After sample digestion, a small aliquot of the solution (Fe ≈ 2500 μg; Ni ≈ 200 μg) was taken for Fe and Ni isotopic analyses. The aliquot was taken to dryness and re‐dissolved in 0.2 ml 10.5M HCl. Several terrestrial metals were also analyzed in this study, including two NIST Fe‐Ni steels (SRM 126c and SRM 361) and two naturally occurring Fe‐Ni alloys (josephinite and awaruite); the josephinite is from Josephine Creek in Oregon (USA), and the awaruite sample is from Big Bay (New Zealand). Terrestrial metals were digested in 3 ml 2:1 concentrated HNO_3_‐HCl in Teflon beakers at 130 °C. The solutions were dried, re‐fluxed in concentrated HCl at 100 °C, dried again, and re‐dissolved in 0.2 ml 10.5M HCl for column chemistry.

### Separation of Fe and Ni

Iron and Ni were separated by ion exchange resins using two‐column and three‐column protocols, respectively. The separations were based on the work of Kraus and Moore (1953), Cook et al. (2006), and Caletka and Krivan (1983). The samples were first loaded onto BioRad poly‐prep columns filled with 1 ml of anion resin (AG1 × 8, 200‐400 mesh) that had been washed and conditioned with 5 ml MQ, 5 ml 1M HNO_3_, 10 ml MQ, and 5 ml 10.5M HCl. The Ni was collected in the loading fraction along with an additional 1.5 ml 10.5M HCl. The column was then washed with 1.5 ml 10.5M HCl and 5 ml 4M HCl, and Fe was eluted with 5 ml 0.4M HCl. The Fe fraction was dried and re‐dissolved in 0.2 ml 10.5M HCl. To ensure the separation of Fe from the matrix, the above procedure was repeated using a new column and resin.

The Ni fraction from the first column was dried, re‐dissolved in 5 ml 0.2M HCl, and loaded onto a BioRad poly‐prep column filled with 1 ml of cation resin (AG50W × 8, 200‐400 mesh) that had been washed and conditioned with 5 ml MQ, 5 ml 4M HCl, 10 ml MQ, and 5 ml 0.2M HCl. After sample loading, the column was washed with 5 ml 0.2M HCl, 5 ml 1 mM HCl, 6 ml 2M HF, 5 ml 1 mM HCl, 5 ml 0.2M HCl, and 6 ml 1M HCl; nickel was eluted with a further 10 ml 1M HCl. The Ni fraction was dried and re‐dissolved in 2 ml 0.6M HCl‐90% acetone (hereafter HCl‐acetone). The sample was loaded onto a BioRad poly‐prep column filled with 1 ml of cation resin (AG50W × 8, 200‐400 mesh) that had been washed and conditioned with 5 ml MQ, 5 ml 4M HCl, 10 ml MQ, 4 ml 30% MQ‐70% acetone, and 6 ml HCl‐acetone. After sample loading, the column was washed with 10 ml HCl‐acetone, 2 ml 0.2M HCl, and 5 ml 1M HCl; nickel was eluted with an additional 10 ml 1M HCl. The final Fe and Ni fractions were taken to dryness and then dried once in 0.45 ml 2:1 concentrated HNO_3_‐HCl and once in 0.5 ml concentrated HNO_3_. Iron and Ni were then dissolved in ≈0.4M HNO_3_ for isotopic analysis. The yields for Fe and Ni were ≈100%. The full procedural blanks (n = 8) were ≈16 ng (Fe) and ≈3 ng (Ni), which are insignificant compared to the amounts of sample Fe and Ni processed.

### Isotopic Analysis of Fe and Ni

Isotopic measurements of Fe and Ni were performed with a ThermoScientific Neptune *Plus* MC‐ICPMS in the Institute for Geochemistry and Petrology (ETH Zürich) in medium resolution mode using standard sampler and skimmer Ni cones. The measurement of Fe isotopes is detailed in Cook and Schönbächler (2017). Briefly, solutions of 10 ppm Fe were introduced with the stable introduction system. All four Fe isotopes (^54^Fe, ^56^Fe, ^57^Fe, ^58^Fe) were measured simultaneously in static mode along with ^53^Cr and ^60^Ni, which were used to correct interferences on ^54^Fe from ^54^Cr and on ^58^Fe from ^58^Ni. The ^56^Fe signal was measured using a 10^10^ Ohm resistor, and the ^53^Cr and ^60^Ni signals were measured using 10^12^ Ohm resistors. The instrumental mass bias was corrected using the exponential law (e.g., Hart and Zindler 1989; Albarède et al. 2004) with ^57^Fe/^54^Fe = 0.36255 (Taylor et al. 1992). For Ni analyses, all five Ni isotopes (^58^Ni, ^60^Ni, ^61^Ni, ^62^Ni, ^64^Ni) were measured simultaneously in static mode along with ^57^Fe and ^66^Zn, which were used to correct interferences on ^58^Ni from ^58^Fe and on ^64^Ni from ^64^Zn. The ^58^Ni signal was measured using a 10^10^ Ohm resistor, and the ^57^Fe and ^66^Zn signals were measured using 10^12^ Ohm resistors. Sample solutions of 3 ppm Ni were introduced using an Aridus II with an uptake rate of 100 μL/min, which provided beams corresponding to a signal of ≈ 75 V on the most abundant isotope (i.e., ^58^Ni). Instrumental mass bias was corrected using the exponential law with either ^61^Ni/^58^Ni = 0.0167442 or ^62^Ni/^58^Ni = 0.0533886 (Gramlich et al. 1989). All sample analyses (Fe and Ni) consisted of 15 measurements (1 measurement = 20 integrations of 8.4 s) bracketed by measurements of the IRMM‐014 (Fe) or SRM 986 (Ni) isotopic standards. A washout time of 210s was used after each measurement. Each analytical session was preceded by a single 300s measurement of the electronic baseline, which was subtracted from all signal intensities. For each sample (terrestrial or iron meteorite) measurement, the isotopic ratios are calculated as the parts per 10,000 deviation relative to the mean values of the two bracketing standards surrounding the measurement of the sample and are reported using the epsilon notation (i.e., ε^i^Fe or ε^i^Ni). Uncertainties reported for individual samples (Tables 1 and 2) represent 95% confidence intervals based on the 15 repeat measurements of each sample solution and were calculated using the following equation:

$$95\%CI=\sqrt{\frac{1}{n-1}\sum_{k=1}^{n}{\left(\varepsilon_{k}-\bar{\varepsilon }\right)}^{2}}\times \frac{t_{0.95}}{\sqrt{n}}\;\left(1\right)$$

where $\bar{\varepsilon }$ is the mean value of the 15 repeats and *t*_0.95_ is Student's *t*‐value for a two‐sided 95% confidence interval with *n*‐1 degrees of freedom (e.g., Qin et al. 2008).

**Table 1:** Nickel and Fe isotopic compositions of terrestrial Fe‐Ni alloys.

Sample	ε^60^Ni (61/58) ± 2SE	ε^62^Ni (61/58) ± 2SE	ε^64^Ni (61/58) ± 2SE	ε^60^Ni (62/58) ± 2SE	ε^61^Ni (62/58) ± 2SE	ε^64^Ni (62/58) ± 2SE	ε^56^Fe ± 2SE	ε^58^Fe ± 2SE
Josephinite	−0.05 ± 0.04	0.06 ± 0.10	0.24 ± 0.12	−0.07 ± 0.04	−0.04 ± 0.07	0.14 ± 0.14	0.00 ± 0.04	−0.05 ± 0.20
Awaruite	−0.01 ± 0.05	0.00 ± 0.11	0.19 ± 0.20	−0.01 ± 0.02	0.00 ± 0.08	0.21 ± 0.11	−0.01 ± 0.03	0.00 ± 0.18
SRM 361	−0.01 ± 0.06	0.15 ± 0.13	0.12 ± 0.15	−0.08 ± 0.03	−0.11 ± 0.10	−0.08 ± 0.10	0.01 ± 0.03	0.04 ± 0.16
SRM 126c‐1	0.01 ± 0.04	0.01 ± 0.10	0.04 ± 0.11	0.00 ± 0.04	0.00 ± 0.07	0.04 ± 0.10	0.04 ± 0.03	−0.05 ± 0.14
SRM 126c‐2	0.01 ± 0.04	0.05 ± 0.11	012 ± 0.15	0.00 ± 0.03	−0.04 ± 0.08	0.07 ± 0.08	0.01 ± 0.04	0.06 ± 0.24
SRM 126c‐3	−0.01 ± 0.06	0.02 ± 0.12	0.02 ± 0.15	−0.01 ± 0.03	−0.01 ± 0.09	−0.04 ± 0.13	−0.01 ± 0.05	−0.13 ± 0.09
SRM 126c‐4	−0.02 ± 0.04	−0.04 ± 0.12	−0.08 ± 0.18	0.01 ± 0.05	0.03 ± 0.09	−0.04 ± 0.13	−0.02 ± 0.04	0.05 ± 0.14
SRM 126c‐5	0.01 ± 0.05	0.04 ± 0.12	0.08 ± 0.18	−0.01 ± 0.04	−0.03 ± 0.09	0.00 ± 0.11	0.00 ± 0.02	−0.14 ± 0.13
SRM 126c‐6	−0.01 ± 0.04	−0.01 ± 0.09	0.02 ± 0.16	−0.01 ± 0.02	0.01 ± 0.07	0.04 ± 0.09	0.02 ± 0.05	−0.14 ± 0.15
SRM 126c‐7	−0.05 ± 0.03	−0.04 ± 0.09	−0.04 ± 0.10	−0.01 ± 0.02	0.03 ± 0.07	0.07 ± 0.08	0.03 ± 0.03	−0.04 ± 0.12
SRM 126c‐8	0.01 ± 0.03	0.04 ± 0.09	0.07 ± 0.12	−0.01 ± 0.02	−0.03 ± 0.07	0.01 ± 0.07	0.00 ± 0.03	0.13 ± 0.13
SRM 126c‐9	−0.01 ± 0.03	0.04 ± 0.05	0.05 ± 0.12	−0.03 ± 0.02	−0.03 ± 0.04	0.00 ± 0.08	0.01 ± 0.03	−0.19 ± 0.22
SRM 126c‐10	0.02 ± 0.04	0.05 ± 0.08	0.15 ± 0.15	−0.01 ± 0.02	−0.04 ± 0.06	0.04 ± 0.11	0.03 ± 0.03	0.04 ± 0.12
SRM 126c‐11	0.05 ± 0.06	0.07 ± 0.12	0.26 ± 0.13	0.02 ± 0.02	−0.06 ± 0.09	0.14 ± 0.09	0.03 ± 0.04	0.04 ± 0.14
SRM 126c‐12	−0.01 ± 0.04	−0.03 ± 0.07	0.00 ± 0.18	0.01 ± 0.02	0.02 ± 0.05	0.04 ± 0.10	0.01 ± 0.04	−0.01 ± 0.14
SRM 126c‐13	0.05 ± 0.05	0.18 ± 0.11	0.21 ± 0.16	−0.05 ± 0.02	−0.13 ± 0.09	−0.06 ± 0.10	0.00 ± 0.06	0.09 ± 0.18
SRM 126c‐14	−0.05 ± 0.04	−0.04 ± 0.10	0.05 ± 0.10	−0.03 ± 0.02	0.03 ± 0.08	0.11 ± 0.11	0.03 ± 0.03	0.00 ± 0.16
SRM 126c‐15	−0.02 ± 0.04	−0.02 ± 0.07	0.03 ± 0.09	−0.01 ± 0.02	0.02 ± 0.05	0.04 ± 0.06	0.01 ± 0.05	−0.01 ± 0.16
SRM 126c‐16	0.00 ± 0.04	0.02 ± 0.09	0.09 ± 0.13	−0.01 ± 0.02	−0.01 ± 0.07	0.06 ± 0.04	0.01 ± 0.03	0.01 ± 0.10
SRM 126c‐17	0.00 ± 0.04	0.02 ± 0.07	0.08 ± 0.13	−0.02 ± 0.03	−0.02 ± 0.05	0.04 ± 0.08	0.02 ± 0.02	−0.03 ± 0.09
SRM 126c‐18	0.00 ± 0.04	0.04 ± 0.08	0.07 ± 0.12	−0.01 ± 0.02	−0.02 ± 0.06	0.02 ± 0.07	0.01 ± 0.03	−0.16 ± 0.12
SRM 126c‐19	0.00 ± 0.04	0.04 ± 0.06	0.14 ± 0.10	−0.02 ± 0.02	−0.03 ± 0.05	0.06 ± 0.06	0.03 ± 0.03	0.00 ± 0.18
Mean ± 2SD	0.00 ± 0.05	0.03 ± 0.11	0.09 ± 0.17	−0.02 ± 0.05	−0.02 ± 0.08	0.04 ± 0.14	0.01 ± 0.03	−0.02 ± 0.17

Values are expressed relative to SRM 986 (Ni) or IRMM‐014 (Fe) in parts per 10,000. The notation “(61/58)” and “(62/58)” indicates the ratio used for the mass bias correction (e.g.*,* ^61^Ni/^58^Ni). SE represents the 95% confidence interval for the individual measurements.

**Table 2:** Nickel and Fe isotopic compositions of IAB iron meteorites.

Sample	ε^60^Ni (61/58) ± 2SE	ε^62^Ni (61/58) ± 2SE	ε^64^Ni (61/58) ± 2SE	ε^60^Ni (62/58) ± 2SE	ε^61^Ni (62/58) ± 2SE	ε^64^Ni (62/58) ± 2SE	ε^56^Fe ± 2SE	ε^58^Fe ± 2SE
Caddo County	−0.05 ± 0.04	−0.11 ± 0.07	−0.17 ± 0.13	0.01 ± 0.02	0.08 ± 0.05	−0.02 ± 0.08	0.02 ± 0.03	−0.09 ± 0.17
Canyon Diablo	0.01 ± 0.04	0.03 ± 0.07	0.00 ± 0.12	−0.01 ± 0.02	−0.02 ± 0.06	−0.01 ± 0.07	0.03 ± 0.03	−0.10 ± 0.13
Cranbourne	−0.06 ± 0.04	−0.05 ± 0.09	−0.10 ± 0.17	−0.01 ± 0.02	0.04 ± 0.07	−0.01 ± 0.09	−0.02 ± 0.03	−0.05 ± 0.10
Magura	−0.04 ± 0.04	−0.12 ± 0.06	−0.11 ± 0.12	0.02 ± 0.02	0.09 ± 0.04	0.04 ± 0.06	−0.01 ± 0.02	0.08 ± 0.17
Odessa	−0.04 ± 0.05	−0.06 ± 0.10	−0.11 ± 0.15	−0.01 ± 0.02	0.05 ± 0.08	−0.02 ± 0.07	−0.01 ± 0.04	−0.05 ± 0.10
Toluca	−0.02 ± 0.03	0.00 ± 0.05	0.00 ± 0.09	−0.02 ± 0.01	0.00 ± 0.04	0.00 ± 0.07	−0.01 ± 0.03	−0.05 ± 0.19
Mean ± 2SE	−0.03 ± 0.03	−0.05 ± 0.06	−0.08 ± 0.07	−0.01 ± 0.02	0.04 ± 0.05	0.00 ± 0.02	0.00 ± 0.02	−0.04 ± 0.06

Values are expressed relative to SRM 986 (Ni) or IRMM‐014 (Fe) in parts per 10,000. The notation “(61/58)” and “(62/58)” indicate the ratio used for the mass bias correction (e.g.*,* ^61^Ni/^58^Ni). SE represents the 95% confidence interval for the individual measurements and the group IAB means.

### Model of Cosmogenic Production Rates for Fe and Ni Isotopes

The cosmogenic production rates of Fe and Ni isotopes have been calculated using the model of Leya and Masarik (2013). In brief, the production rates were calculated using the excitation functions of the relevant nuclear reactions and the particle spectra for primary and secondary particles. The model includes proton‐ and neutron‐induced spallation reactions on Fe and Ni (for the production of Fe isotopes) and on Ni (for the production of Ni isotopes). The excitation functions were calculated using the TALYS‐1.8 code (Koning et al. 2015) because no experimental cross sections are available for the relevant reactions. TALYS is limited to projectile energies below 240 MeV; thus, to cover the full energy range, we used the new version of INCL for projectiles with higher energies (up to 20 GeV). In addition to the spallation reactions, we considered neutron capture reactions on Fe and Ni (i.e., production and burnout of Fe and Ni isotopes), as well as neutron capture on ^55^Mn to produce ^56^Fe, on ^59^Co to produce ^60^Ni, and on ^63^Cu to produce ^64^Ni. The cross sections for thermal and epithermal neutron capture reactions were taken from the JEFF‐3.0A database (Santamarina et al. 2009).

The particle spectra for primary and secondary particles are the same as described in Leya and Masarik (2013). The spectra were calculated using the LAHET code (Prael and Lichtenstein 1989) by following the trajectories of primary and secondary particles. We assume a primary GCR particle flux of 2.99 cm^−2^ s^−1^ (Kollár et al. 2006) for calculating neutron capture rates and 4.47 cm^−2^ s^−1^ (e.g., Ammon et al. 2009; Leya and Masarik 2013) for spallation reactions. The modeled production rates are a function of the pre‐atmospheric radius of the meteoroid, assumed to be spherical, and the depth below the surface. Iron and Ni isotopic ratios were calculated for a range of shielding conditions (i.e., radii from 5 to 120 cm) by assuming a single‐stage irradiation for a given exposure age and chemical composition.

## Results

### Terrestrial Fe‐Ni Alloys

Several terrestrial Fe‐Ni alloys were analyzed to validate the overall method for Fe and Ni analyses. They consisted of one sample each of NIST SRM 361 (high‐Ni steel), josephinite, and awaruite. In addition, 19 aliquots of NIST SRM 126c (steel) were processed through the entire separation protocol (Table 1). In aggregate, these 22 samples define external reproducibilities (2SD) of ε^56^Fe ± 0.03, ε^58^Fe ± 0.17, ε^60^Ni (61/58) ± 0.05, ε^62^Ni (61/58) ± 0.11, ε^64^Ni (61/58) ± 0.17, ε^60^Ni (62/58) ± 0.05, ε^61^Ni (62/58) ± 0.08, ε^64^Ni (62/58) ± 0.14. The notations “(61/58)” and “(62/58)” indicate the ratio used for the mass bias correction. After chemical separation, all terrestrial Fe fractions had Ni/Fe ≤ 3.1 × 10^−5^ and Cr/Fe ≤ 9.5 × 10^−7^; the Ni fractions had Fe/Ni ≤ 2.7 × 10^−4^ and Zn/Ni ≤ 1.1 × 10^−5^. These ratios are at the levels required to make accurate isotopic measurements of Fe and Ni in the presence of isobaric interferences (e.g., Cook et al. 2006; Dauphas et al. 2009; Steele et al. 2011). For Ni isotopes, the overall mean values for ε^60^Ni (62/58) and ε^64^Ni (61/58) are slightly less accurate than for ε^60^Ni (61/58) and ε^64^Ni (62/58). This may be due to larger differences in the mean mass of the normalizing isotopes relative to the mean mass of those being normalized (Vance and Thirlwall 2002).

### IAB Iron Meteorites

No resolvable variations are observed in ε^56^Fe or ε^58^Fe (Table 2). Some ε^i^Ni values are not within uncertainty of zero, but the individual mean values are within, or near to, the overall analytical resolution. In addition, the group mean values for all six ε^i^Ni values are not resolvable from the terrestrial alloys. After chemical separation, all IAB Fe fractions had Ni/Fe ≤ 2.3 × 10^−5^ and Cr/Fe ≤ 7.7 × 10^−7^; the Ni fractions had Fe/Ni ≤ 1.1 × 10^−4^ and Zn/Ni ≤ 3.8 × 10^−6^; all of these ratios are at the levels required for accurate Fe and Ni isotopic measurements.

## Discussion

Galactic cosmic rays can induce nuclear reactions in extraterrestrial samples prior to their arrival on Earth. These reactions can lead to observable changes in the isotopic composition of the target material if the reactions have a high probability of occurring and/or if the sample is exposed to GCRs for a long period of time. Multiple factors influence the magnitude of GCR effects, including the size of the target, its chemical composition, the duration of exposure, and the depth of the sample below the pre‐atmospheric surface (e.g., Nishiizumi et al. 1986; Masarik and Reedy 1994; Leya et al. 2000). Spallation reactions are largest near the surface because they require high energies, whereas neutron capture reactions peak below the surface because they only occur at low energies (e.g., Reedy and Arnold 1972; Masarik 1997). Consequently, the depth dependency of these reactions differs. Spallation reactions decrease rapidly with depth. Conversely, the flux of low energy neutrons needed for capture reactions increases with depth because scattering events are required to slow down the neutrons; thus, neutron capture reactions reach a maximum at some depth below the surface. For iron meteoroids, the thermal neutron flux peaks near the center of objects with radii around 60–100 cm. Reactions induced by GCRs may lead to production or destruction of particular isotopes. Observed changes in isotopic ratios will depend on whether a net gain or loss of isotopes occurs, as well as how the two isotopes used for the mass bias correction are affected. We first present the results of model predictions for GCR effects on Fe and Ni isotopes in iron meteorites, and then, we use the results for IAB irons to test the predictions. In addition to Fe and Ni isotopes, we use Pt isotopic data from the same samples (Hunt et al. 2018) as a neutron dose monitor to aid comparison of the experimental data to the model. All model results are for an exposure time of 1000 Ma and represent near upper limits on GCR effects because most iron meteorites have CRE ages of ≤ 1000 Ma (e.g., Herzog and Caffee 2014).

### Galactic Cosmic Ray Effects on Fe Isotopes in Iron Meteorites

**Figure 1:**
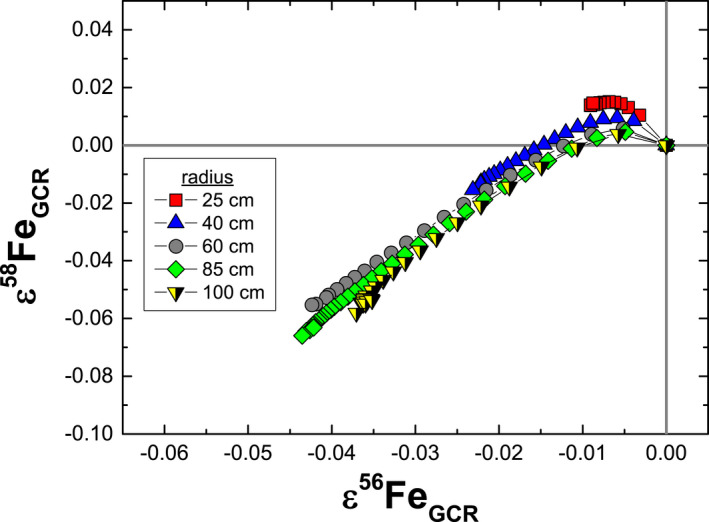
Model results for the combined effects of neutron capture and spallation reactions on ε^58^Fe and ε^56^Fe. The model predictions are for iron meteoroids with various radii, a fixed cosmic ray exposure age of 1000 Ma, and an Ni/Fe ratio of 0.074. This Ni/Fe ratio (Wasson and Kallemeyn 2002) is appropriate for IAB irons.

All four Fe isotopes are affected by spallation and neutron capture reactions. In terms of the mean changes in the relative isotopic abundances, neutron capture reactions lead to a net loss of ^54^Fe and ^56^Fe and a net gain of ^57^Fe and ^58^Fe; the largest effect is on ^57^Fe (2.8 ppm), whereas the effects on the remaining isotopes are all < 0.6 ppm. Spallation reactions lead to a net gain of ^54^Fe and a net loss of ^56^Fe, ^57^Fe, and ^58^Fe; the largest effect is on ^54^Fe (0.17 ppm), whereas the effects on the remaining isotopes are all < 0.02 ppm. Changes in isotopic ratios are then a combination of the total GCR effects (neutron capture and spallation) on the individual isotopes, as well as how the effects on the normalizing ratio (e.g., ^57^Fe/^54^Fe) propagate through the mass bias correction. For the mass bias‐corrected isotopic ratios, neutron capture reactions lead to deficits in both ε^56^Fe and ε^58^Fe. However, for an exposure of 1000 Ma, the predicted effects are small: ε^56^Fe ≤ −0.043 and ε^58^Fe ≤ −0.071. In addition to the neutron capture and spallation reactions on Fe, we also considered neutron capture on Mn, which can produce ^56^Fe via the reaction pathway ^55^Mn + n → ^56^Mn → ^56^Fe + β^−^. However, at the extremely low Mn/Fe ratios in iron meteorites (Walker et al. 2008; McCoy et al. 2011), the effects of this reaction are insignificant (<0.0001 ε). Spallation of Ni isotopes leads to an excess in ε^58^Fe. Of the iron meteorite groups, the IVB irons are the most Ni rich; the maximum probable spallation effect for an Ni/Fe ratio of 0.2 (Walker et al. 2008) is ≤0.065, which competes with the deficits caused by neutron capture. Spallation effects on ε^56^Fe are insignificant (≤−0.007). The combined effects of neutron capture and spallation (Fig. 1) show that neutron capture dominates the effects on both ε^56^Fe and ε^58^Fe. Nearly all the GCR effects on ε^56^Fe and ε^58^Fe are smaller than the analytical resolution; only the most extreme ε^56^Fe deficits produced by GCRs are larger. Although the target composition is not the same (alloy versus sulfide), the above results suggest that the ε^56^Fe deficits observed earlier in some FeS inclusions from iron meteorites (Cook and Schönbächler 2017) are unlikely to result entirely from GCR effects. The particle spectra for iron meteorites are completely dominated by the bulk chemical composition (i.e., Fe and Ni). Therefore, our model predictions are also applicable to FeS inclusions in iron meteorites, so long as they occur as a trace mineral phase. Note that neither spallation nor neutron capture reactions on S can produce Fe or Ni isotopes. Based on our model, the resolved ε^56^Fe deficits in FeS reported by Cook and Schönbächler (2017) are too large to explain solely as GCR effects, and an additional/alternative origin is required.

### Galactic Cosmic Ray Effects on Ni Isotopes in Iron Meteorites

Similar to Fe isotopes, all five Ni isotopes are affected by spallation and neutron capture reactions. In terms of the mean changes in the relative isotopic abundances, neutron capture reactions lead to a net loss of ^58^Ni and ^62^Ni and a net gain of ^60^Ni, ^61^Ni, and ^64^Ni; the largest effect is on ^61^Ni (1.7 ppm), whereas the effects on the remaining isotopes are all <0.2 ppm. The magnitude of the change in the ^62^Ni abundance agrees with the estimate by Chen et al. (2016). Spallation reactions lead to only very small changes in the five Ni isotopes; the largest effect is on ^61^Ni (≈0.3 ppm), whereas the effects on the remaining isotopes are all <0.01 ppm. For the mass bias‐corrected isotopic ratios, neutron capture reactions lead to small excess in ε^61^Ni (62/58) ≤0.039 and ε^64^Ni (62/58) ≤0.0053 and small deficits in ε^62^Ni (61/58) ≤−0.052 and ε^64^Ni (61/58) ≤−0.073. In addition to neutron capture reactions on Ni, neutron capture reactions on Cu also contribute to ^64^Ni production via the reaction pathway ^63^Cu + n → ^64^Cu → ^64^Ni + β^+^ (19%). Unstable ^64^Cu may also decay to ^64^Ni by electron capture (41%). The remaining ^64^Cu (40%) decays to ^64^Zn + β^−^ (Friedlander et al. 1981). In general, Cu/Ni ratios in irons are low and range from ≈0.002 to ≈0.003 (Wasson 1999; Wasson and Richardson 2001; Wasson and Kallemeyn 2002; Wasson and Huber 2006; Wasson et al. 2007; Wasson and Choe 2009). For a CRE age of 1000 Ma, a Cu/Ni ratio of 0.003 leads to an additional maximum increase in ε^64^Ni values by <0.001. Thus, the contribution to ^64^Ni production by neutron capture on ^63^Cu is not significant and is not sensitive to the small Cu/Ni ratios in irons. Conversely, the effects on ε^60^Ni are sensitive to the Co/Ni ratio; neutron capture on Co produces ^60^Ni via the reaction pathway ^59^Co + n → ^60^Co → ^60^Ni + β^−^. In iron meteorites, Co/Ni ratios range from ≈0.04 to ≈0.12 (Scott 1977; Malvin et al. 1984; Wasson 1999; Wasson and Richardson 2001; Wasson and Kallemeyn 2002; Wasson and Huber 2006, Wasson et al. 2007; Walker et al. 2008; Wasson and Choe 2009). The ε^60^Ni (62/58) offsets are only positive and range from 0.016 to 0.044, whereas the ε^60^Ni (61/58) values may be negative or positive and range from −0.012 to 0.019.

**Figure 2:**
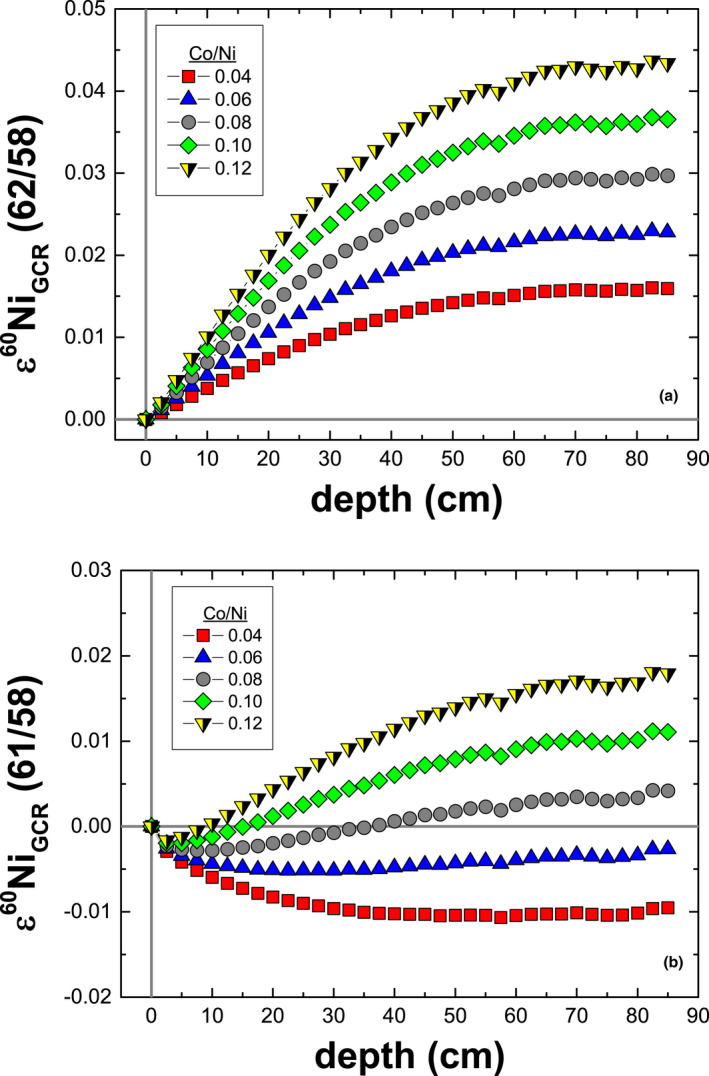
Model results for the combined effects of neutron capture and spallation reactions on (a) ε^60^Ni (62/58) and (b) ε^60^Ni (61/58). The model predictions are for iron meteoroids with a fixed radius of 85 cm and cosmic ray exposure age of 1000 Ma but with variable Co/Ni ratios.

**Figure 3:**
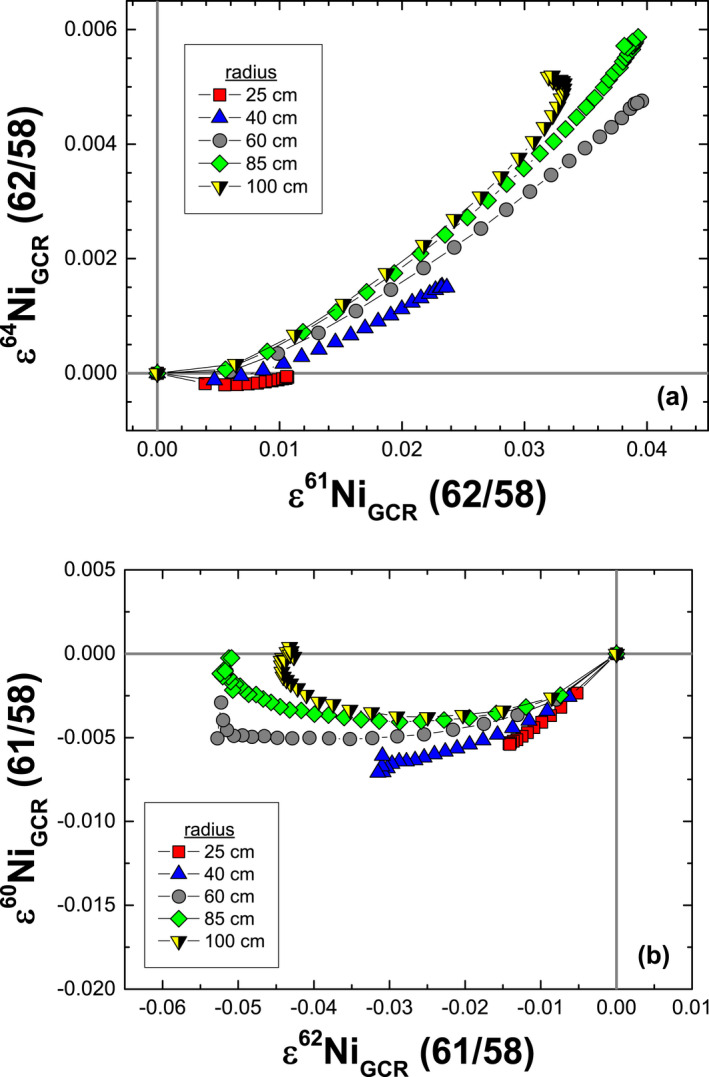
Model results for the combined effects of neutron capture and spallation reactions on (a) ε^64^Ni (62/58) versus ε^61^Ni (62/58) and (b) ε^60^Ni (61/58) versus ε^62^Ni (61/58). The model predictions are for iron meteoroids with various radii, a fixed cosmic ray exposure age of 1000 Ma, a Co/Ni ratio of 0.067, and a Cu/Ni ratio of 0.0022. The Co/Ni and Cu/Ni ratios (Wasson and Kallemeyn 2002) are appropriate for IAB irons.

The effects from spallation reactions on all ε^i^Ni values are smaller than those from neutron capture and are <0.01 ε, with the largest effects on ε^64^Ni (62/58) ≤−0.0087. For all ε^i^Ni values, the combined neutron capture and spallation effects are smaller than the analytical resolution. Choosing a normalizing ratio that minimizes the effects of GCRs will aid in the search for small nucleosynthetic variations in isotopic ratios. The combined effects on ε^60^Ni are minimized when using ε^60^Ni (61/58; Fig. 2). The combined GCR effects for other selected ε^i^Ni values (Fig. 3) are minimized using the (62/58) normalization scheme. For example, the ε^64^Ni (62/58) offsets are an order of magnitude smaller than for ε^64^Ni (61/58)—not shown. The effects on ε^61^Ni (62/58) and ε^62^Ni (61/58) are of similar magnitudes but opposite in direction. For a Co/Ni ratio appropriate for IAB irons (Wasson and Kallemeyn 2002), the GCR effects on ε^60^Ni (61/58) are insignificant (≤ −0.0071).

### Comparing IAB Irons to GCR Model Predictions

**Figure 4:**
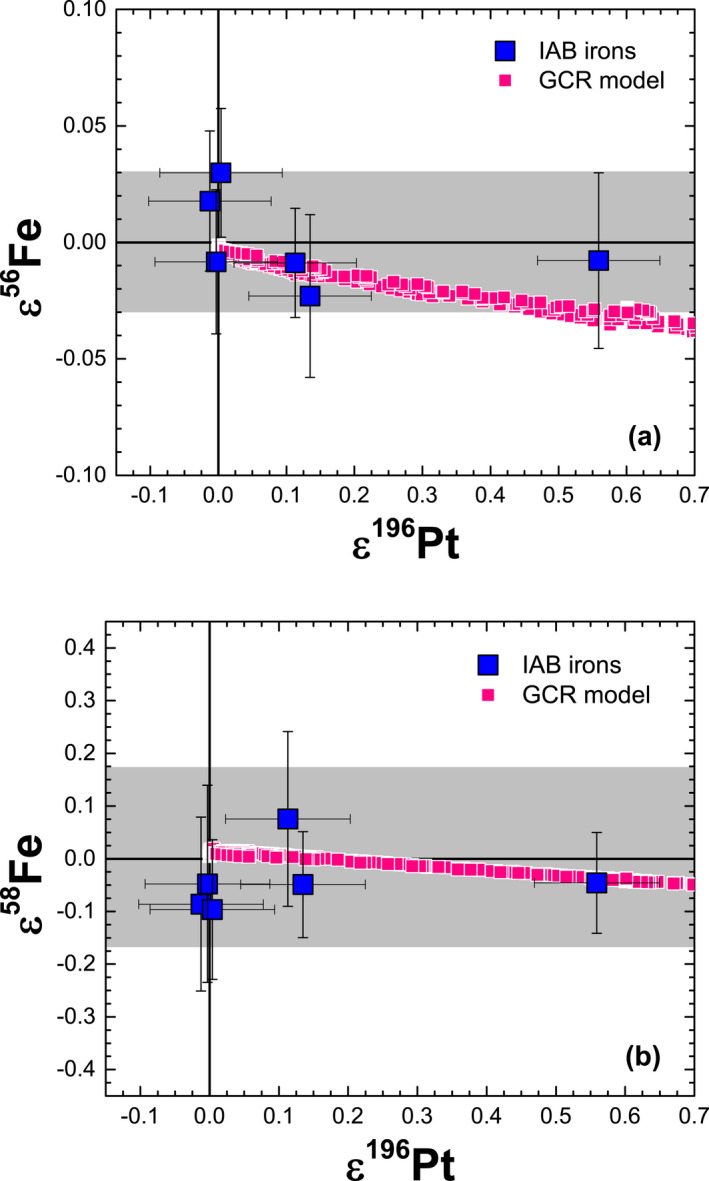
Plots of (a) ε^56^Fe versus ε^196^Pt and (b) ε^58^Fe versus ε^196^Pt measured in IAB iron meteorites along with galactic cosmic ray model results, which represent meteoroids with radii spanning 5–120 cm, a cosmic ray exposure age of 1000 Ma, and an Ni/Fe ratio of 0.074 (Wasson and Kallemeyn 2002). The gray band represents the analytical resolution for ε^i^Fe, based on data for terrestrial samples. Data for ε^196^Pt are from Hunt et al. (2018).

**Figure 5:**
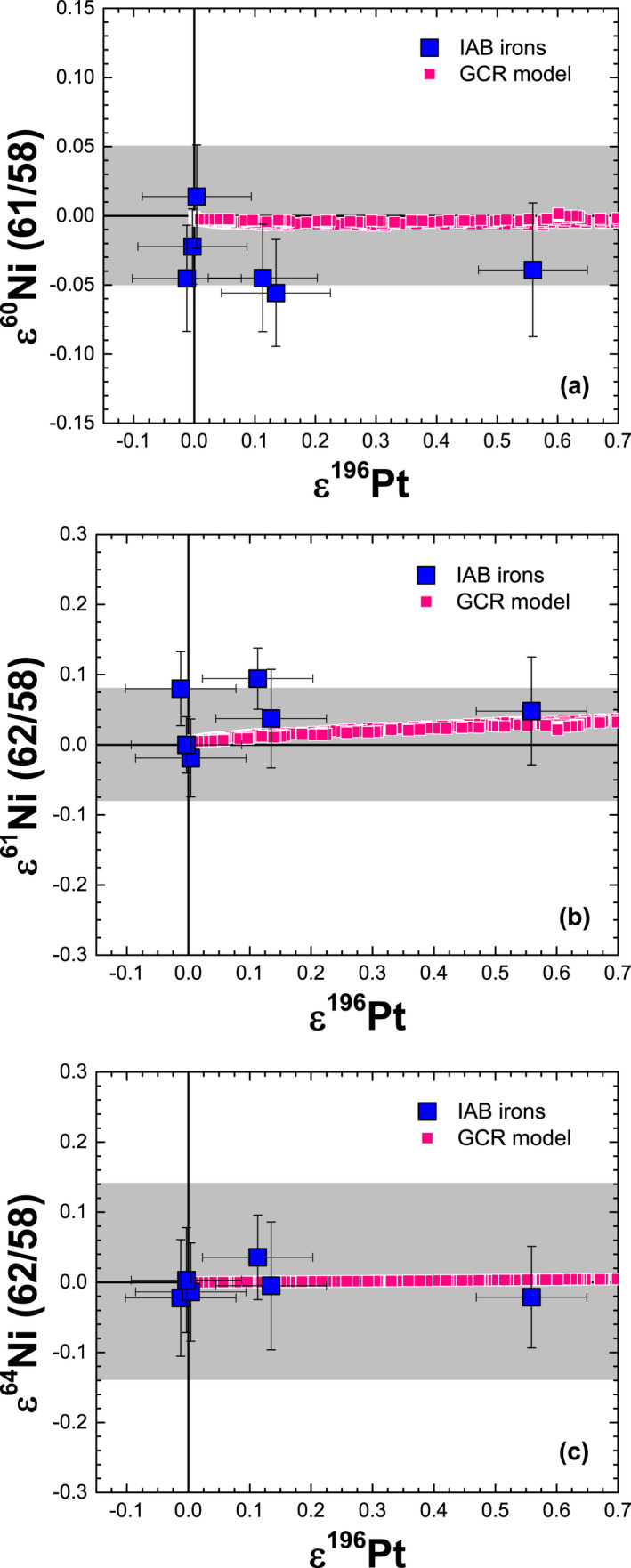
Plots of (a) ε^60^Ni (61/58) versus ε^196^Pt, (b) ε^61^Ni (62/58) versus ε^196^Pt, and (c) ε^64^Ni (62/58) versus ε^196^Pt measured in IAB iron meteorites along with galactic cosmic ray model results, which represent meteoroids with radii spanning 5–120 cm, a cosmic ray exposure age of 1000 Ma, a Co/Ni ratio of 0.067, and a Cu/Ni ratio of 0.0022 (Wasson and Kallemeyn 2002). The gray band represents the analytical resolution for ε^i^Ni, based on data for terrestrial samples. Data for ε^196^Pt are from Hunt et al. (2018).

Platinum isotopes have been shown to be a reliable proxy for neutron capture effects in iron meteorites (e.g., Kruijer et al. 2013; Wittig et al. 2013; Hunt et al. 2018). Except for some ε^58^Fe values, our GCR model predicts that spallation effects on Fe and Ni isotopic ratios are always <0.01 ε; hence, we plot our ε^i^Fe and ε^i^Ni values versus those for ε^i^Pt measured in the same samples (Hunt et al. 2018) to test for consistency between the GCR model and the experimental data for IAB irons. For our samples, three IAB irons (Caddo County, Canyon Diablo, Toluca) show no resolvable GCR effects in Pt isotopes (i.e., ε^196^Pt ≈ 0), two (Cranbourne, Magura) show small offsets due to GCR effects, and one (Odessa) shows large GCR effects of ≈0.6 (Hunt et al. 2018). Many iron meteorites have ε^196^Pt values ≤0.5 (e.g., Wittig et al. 2013; Kruijer et al. 2014; Nanne et al. 2019). Thus, our sample suite represents a broad range of exposure histories. Figures 4 and 5 show ε^i^Fe and ε^i^Ni values versus those for ε^196^Pt. In all cases, the measured ε^i^Fe and ε^i^Ni values in IAB irons are in good agreement with the GCR model predictions and do not show resolvable variations, despite the large effects observed on ε^196^Pt in Odessa. The remaining ε^i^Ni values are in equally good agreement with the GCR model predictions as those shown in Fig. 5.

### Model Predictions for GCR Effects in Chondrites

Although the focus of the current study is on iron meteorites, we briefly discuss the model predictions for GCR‐induced effects on Fe and Ni isotopes in chondrites (carbonaceous and ordinary). The relevant elemental ratios (i.e., Mn/Fe, Ni/Fe, Co/Ni) show limited variations among the chondrite groups. For modelling purposes, we input the ratios for CI carbonaceous chondrites (Lodders 2003) and the mean values (H, L, LL) for ordinary chondrites (Wasson and Kallemeyn 1988). In addition, we used a CRE age of 100 Ma; thus, the model results represent upper limits on GCR effects because carbonaceous and ordinary chondrites have CRE ages <100 Ma (Herzog and Caffee 2014). As for iron meteorites, neutron capture effects dominate over spallation effects in chondrites. The maximum combined effects on Fe isotopes in both chondrite classes are ε^56^Fe <−0.018 and ε^58^Fe <−0.030. Modeled offsets for ε^60^Ni (61/58) and ε^60^Ni (62/58) are less than ±0.010. The predicted effects on all other ε^i^Ni values are less than ±0.033. Therefore, no significant GCR‐induced effects are expected on ε^i^Fe or ε^i^Ni values in carbonaceous or ordinary chondrites.

## Conclusions

We present model results of cosmogenic production rates to quantify the potential GCR effects on Fe and Ni isotopes in iron meteorites. The model reveals that neutron capture reactions dominate over spallation reactions for both Fe and Ni isotopes. Except for the largest deficits in ε^56^Fe, the total combined GCR effects on ε^i^Fe and ε^i^Ni values are less than the analytical resolution, and most effects are negligible. The GCR effects on ^60^Ni and ^64^Ni can be minimized by using ε^60^Ni (61/58) and ε^64^Ni (62/58), which also show better accuracy compared to the alternative normalizations. Iron and Ni isotopic data for a suite of six group IAB irons are consistent with the lack of resolvable GCR‐induced variations predicted by the model. The minimal effects of GCRs on Fe and Ni isotopes should not hamper the search for nucleosynthetic variations or the application of the ^60^Fe‐^60^Ni chronometer in iron meteorites or chondrites. However, for samples that have been strongly irradiated, it should be possible to apply a correction, if necessary, using the GCR model in combination with an independent neutron dosimeter like Pt (or Os). Our GCR model results for εFe and εNi values are highly correlated with those modeled for ε^196^Pt. A potential GCR correction could be beneficial for ε^56^Fe deficits that exceed the analytical precision or for ε^60^Ni (62/58) values in samples with elevated Co/Ni ratios.

## Editorial Handling

Dr. A. J. Timothy Jull
